# Epidemiological changes of *Mycoplasma pneumoniae* among children before, during, and post the COVID-19 pandemic in Henan, China, from 2017 to 2024

**DOI:** 10.1128/spectrum.03121-24

**Published:** 2025-06-09

**Authors:** Jiahui Qi, Hongwei Li, Hao Li, Zhuyuan Wang, Shuo Zhang, Xiangying Meng, Xin Zhao, Enwu Yuan, Linlin Zhang

**Affiliations:** 1 Department of Laboratory Medicine, the Third Affiliated Hospital of Zhengzhou University 647990, Zhengzhou, Henan, China; 2 Zhengzhou Key Laboratory for In Vitro Diagnosis of Hypertensive Disorders of Pregnancy, Zhengzhou, Henan, China; 3 Tianjian Laboratory of Advanced Biomedical Sciences, Institute of Advanced Biomedical Sciences, Zhengzhou University, Zhengzhou, Henan, China; Institut National de Santé Publique du Québec, Sainte-Anne-de-Bellevue, Québec, Canada

**Keywords:** *Mycoplasma pneumoniae*, children, COVID-19, macrolide-resistant mutations of *M. pneumoniae*, nonpharmaceutical interventions

## Abstract

Studies characterizing the epidemic of *Mycoplasma pneumoniae* (*M. pneumoniae*) in Henan Province are scarce. Our research investigated the epidemiological changes of *M. pneumoniae* and macrolide-resistant mutations of *M. pneumonia*e (MRMP) among children under 18 years from January 2017 to December 2024. The study was categorized into three stages based on the COVID-19 timeline (pre-pandemic, pandemic, and post-pandemic) to analyze the impact of nonpharmaceutical interventions (NPIs) on *M. pneumoniae* in Henan Province. Among 27,056 children, 28.36% tested positive for *M. pneumoniae*, with the lowest positivity rate in 2022 (12.56%) and the highest in 2023 (35.01%). The positivity rate was greatest in the 6–17 years (41.08%) and lowest in ≤1 year (12.38%). Epidemic seasons were predominantly in autumn (36.71%) and winter (23.12%). The MRMP rate increased from 36.68% in 2018 to 87.16% in 2023, averaging 71.00%. The positivity rate of *M. pneumoniae* was 26.12% during the pre-pandemic stage, significantly declined during the pandemic stage (14.97%), and rebounded to a high level in the post-pandemic stage (31.88%). The MRMP rate also increased across the three stages: 46.74%, 72.63%, and 83.31%, respectively. These results indicate a significant influence of COVID-19 NPIs on the transmission of *M. pneumoniae* and MRMP.

**IMPORTANCE:**

*M. pneumoniae* is a significant cause of community-acquired pneumonia with a high MRMP rate in China. Various NPIs in the COVID-19 pandemic have impacted the prevalence of *M. pneumoniae* and could have important implications for *M. pneumoniae* prevention and control strategies. Our study documented *M. pneumoniae* and MRMP detection before, during, and post the COVID-19 pandemic in Henan, China, from 2017 to 2024, aiming to investigate the impact of NPIs on *M. pneumoniae* transmission and continue to monitor the evolution of the epidemiological characteristics of *M. pneumoniae* infection after the pandemic when restrictive measures are no longer needed.

## INTRODUCTION

*Mycoplasma pneumoniae* (*M. pneumoniae*) is a small organism that lacks a cell wall, grows slowly, and is transmitted primarily through aerosols and droplets. It is a common cause of community-acquired pneumonia (CAP) in children and adolescents worldwide, particularly in school-aged children. Symptoms of *M. pneumoniae* infection include cough, fever, sore throat, headache, and fatigue (1, 2). Epidemics of *M. pneumoniae* can occur at intervals of 3 to 7 years and can last up to 2 years. Infection can occur in any season, with variations in epidemic seasons among different regions (3, 4).

Macrolides inhibit bacterial protein synthesis by targeting 50S ribosomal transpeptidase activity and inducing peptidyl-tRNA dissociation, while exerting anti-inflammatory effects through cytokine suppression and immune modulation, making them pivotal in pediatric *M. pneumoniae* management (5–7). However, since the initial identification of pneumonia attributed to macrolide-resistant *M. pneumoniae* (MRMP) in Japan in 2000, there has been a notable increase in MRMP cases, largely due to the overuse of macrolide antibiotics. These resistant strains frequently result in more severe clinical manifestations, complicating treatment efforts (8). In particular, countries and regions in Asia have markedly elevated rates, with Japan having a prevalence of more than 80% and China reaching a prevalence as high as 99.7% (9, 10).

The global incidence of *M. pneumoniae* verified through direct testing methods was reported to be 8.61% from 2017 to 2020 across all age demographics (11). During the coronavirus disease 2019 (COVID-19) pandemic (2020–2022), a range of large-scale implementations of strict nonpharmaceutical interventions (NPIs), including mask-wearing, social distancing, improving hand hygiene, home isolation, closing recreational venues, adopting remote education, and limiting travel and the “Dynamic COVID-Zero Strategy” in China, were swiftly enacted to mitigate the spread of severe acute respiratory syndrome coronavirus 2 (SARS-CoV-2) (12, 13). Owing to these NPIs, the incidence of *M. pneumoniae* decreased to 1.69% from 2020 to 2021 (11). After December 2022, following the relaxation of the NPI policy in China, noticeable peaks were observed throughout 2023 in the resurgence of respiratory pathogen infections, including influenza viruses, respiratory syncytial virus (RSV), and *M. pneumoniae* (14–16).

However, research focused on the epidemiological characteristics of *M. pneumoniae* infections over a long period within Henan Province, a central region in China, has been relatively limited. This study performed a retrospective examination of data concerning *M. pneumoniae* infections and MRMP over a period of 8 years from 1 January 2017 to 31 December 2024. It includes three distinct stages of the entire COVID-19 timeline: the pre-pandemic stage (2017–2019), the pandemic stage (2020–2022), and the post-pandemic stage (2023–2024). The main aim of this study was to explore the epidemiological features of *M. pneumoniae* and MRMP, as well as to evaluate the influence of NPIs on the epidemiology of *M. pneumoniae* in Henan, thereby contributing to improvements in clinical diagnostic and therapeutic approaches.

## MATERIALS AND METHODS

### Study design

This research was performed at the Third Affiliated Hospital of Zhengzhou University, which is the largest Maternal and Child Health Hospital in Henan Province, China, from 1 January 2017 to 31 December 2024. Participants under 18 years of age who exhibited respiratory infectious diseases (RIDs) such as cough, sore throat, and nasal congestion and were suspected of having an *M. pneumoniae* infection were included. These individuals underwent analysis for *M. pneumoniae* DNA or MRMP tests. Patients who had multiple tests within 3 months were not included in this research. This retrospective study was approved by the Ethics Committee of the Third Affiliated Hospital of Zhengzhou University. Informed consent was waived, as the data collected from medical databases are anonymous, deidentified, and used only for scientific research purposes. All methods were performed in accordance with the relevant guidelines and regulations following the Helsinki Declaration.

### Division of groups

The children were divided into four age categories: ≤1 year, 1–2 years, 3–5 years, and 6–17 years. For the purpose of examining the temporal trends of *M. pneumoniae* infection, this study categorized children into four distinct seasons: spring (March–May), summer (June–August), autumn (September–November), and winter (December–February of the following year). Considering the timeline of COVID-19 (including the implementation and relaxation of NPIs) in Henan Province, the children in this investigation were separated into three stages: stage I (pre-pandemic: 1 January 2017 to 31 December 2019), stage II (pandemic: 1 January 2020 to 31 December 2022), and stage III (post-pandemic: 1 January 2023 to 31 December 2024). These group categories were employed to analyze the data on the *M. pneumoniae* positivity rate and MRMP rate.

### Laboratory testing

From 2017 to 2024, nasopharyngeal swabs (NPS), sputum, and bronchoalveolar lavage fluid (BALF) specimens were collected from pediatric patients for comprehensive *M. pneumoniae* analysis. DNA detection of *M. pneumoniae* was performed using two RT-PCR kits: (a) for the period of 2017–2022, a kit from Amplly Biotech (Xiamen Amplly Biotechnology Co., Xiamen, China) was used, with positivity criteria defined as S-curve amplification and Ct ≤38; (b) for 2023–2024, a kit from Daan Gene (Guangzhou Daan Gene Co., Guangzhou, China) was utilized, with positivity criteria set as S-curve amplification and Ct <30. The MRMP assay (Mole Bioscience, Jiangsu, China) employed the VIC channel to confirm the presence of *M. pneumoniae* DNA (Ct <35) and the FAM channel to detect 23S rRNA mutations A2063G/A2064G (Ct <35). Strains exhibiting dual-signal positivity were classified as macrolide-resistant.

### Statistical analysis

The positivity rates of *M. pneumoniae* and of MRMP across various years, seasons, and months and among patients of different gender and age groups were analyzed via R software (v4.3.3). Pairwise comparisons were made using Pearson’s χ² test with Bonferroni correction in SPSS software version 23 (IBM Corp). A *P*-value < 0.05 was considered statistically significant. A heatmap was generated to illustrate the epidemic distribution of *M. pneumoniae* across different years, where a detection rate of 25% was used as the threshold to indicate an *M. pneumoniae* epidemic. All the results were visualized via the “ggplot2” package (v3.5.0).

## RESULTS

### Basic characteristics of the overall *M. pneumoniae* samples

This study involved a total of 27,056 children between 1 January 2017 and 31 December 2024. Among these participants, 28.36% (7,672/27,056) were positive for *M. pneumoniae*. The infection rates for *M. pneumoniae* were 28.29% (4,229/14,949) for males and 28.44% (3,443/12,107) for females, with no significant difference observed (*P* = 0.787). The median age of the participants was 4.83 years (interquartile range: 2.83–7.00). A total of 41.08% (5,332/12,980) positive tests occurred within the age group of 6–17 years (Table 1).

**TABLE 1:** Basic characteristics of the overall *M. pneumoniae* samples (*n* = 27,056)

Variables	Total (n)	Positive tests (n)	Detection rate (%)^*a*^	Statistic	P
Sex	(*n* = 27,056)			χ²=0.07	0.787
Male	14,949	4,229	28.29		
Female	12,107	3,443	28.44		
Age group, y	(*n* = 27,056)			χ²=2,037.15	<0.001
≤ 1	2,762	342	12.38		
1–2	2,548	363	14.25		
3–5	8,766	1,635	18.65		
6–17	12,980	5,332	41.08		
Season	(*n* = 26,890)			χ²=916.90	<0.001
Spring	3,190	444	13.92		
Summer	3,415	777	22.75		
Autumn	12,578	4,618	36.71		
Winter	7,707	1,782	23.12		

^*a*^ Detection rate (%) was expressed as the positive number/the total number (%).

The classification of cases revealed 4,119 cases (15.22%, 4,119/27,056) at stage I, 4,235 cases (15.65%, 4,235/27,056) at stage II, and 18,702 cases (69.12%, 18,702/27,056) as stage III, with positivity rates for *M. pneumoniae* of 26.12%, 14.97%, and 31.88%, respectively (Table 2).

**TABLE 2:** Comparison of the positive rate of *M. pneumoniae* in three stages according to the COVID-19 epidemic status^*a*^

Group	Stage I (2017–2019)		Stage II (2020–2022)		Stage III (2023–2024)		Stage I vs Stage II		Stage II vs Stage III		Stage I vs Stage III	
	No. Pos/no. Tested	Detection rate, %^*a*^	No. Pos/no. Tested	Detection rate, %^*a*^	No. Pos/no. Tested	Detection rate, %^*a*^	Statistic	*P* value	Statistic	*P* value	Statistic	*P* value
PCR testing												
Overall	1,076/4,119	26.12	634/4,235	14.97	5,962/18,702	31.88	χ²=159.53	<0.001	χ²=481.87	<0.001	χ²=52.43	<0.001
Age group, y												
≤ 1	109/640	17.03	42/509	8.25	191/1,613	11.84	χ²=19.15	<0.001	χ²=5.10	0.024	χ²=10.69	0.001
1–2	104/626	16.61	49/577	8.49	210/1,345	15.61	χ²=17.84	<0.001	χ²=17.56	<0.001	χ²=0.32	0.572
3–5	388/1,790	21.68	196/1,942	10.09	1,051/5,034	20.88	χ²=94.68	<0.001	χ²=111.04	<0.001	χ²=0.51	0.477
6–17	475/1,063	44.68	347/1,207	28.75	4,510/10,710	42.11	χ²=62.14	<0.001	χ²=80.20	<0.001	χ²=2.63	0.105
Gender												
Male	603/2,328	25.90	351/2,385	14.72	3,275/10,236	31.99	χ²=91.29	<0.001	χ²=282.01	<0.001	χ²=33.00	<0.001
Female	473/1,791	26.41	283/1,850	15.30	2,687/8,466	31.74	χ²=68.30	<0.001	χ²=200.19	<0.001	χ²=19.69	<0.001
Season												
Spring	152/862	17.63	96/834	11.51	196/1,494	13.12	χ²=12.73	<0.001	χ²=1.26	0.261	χ²=8.85	0.003
Summer	224/794	28.21	195/1,030	18.93	358/1,591	22.50	χ²=21.82	<0.001	χ²=4.79	0.029	χ²=9.36	0.002
Autumn	431/1,223	35.24	160/1,057	15.14	4,027/10,298	39.10	χ²=119.34	<0.001	χ²=236.57	<0.001	χ²=6.88	0.009
Winter	218/1,074	20.30	183/1,314	13.93	1,381/5,319	25.96	χ²=17.17	<0.001	χ²=84.72	<0.001	χ²=15.29	<0.001

^*a*^ Detection rate (%) was expressed as the positive number/the total number (%), The Bonferroni method was used for pairwise comparisons, *P* < 0.05/3, which corresponds to *P* < 0.0167 was considered statistical significant.

### Temporal trends and seasonality of *M. pneumoniae* from 2017 to 2024

**Fig 1:**
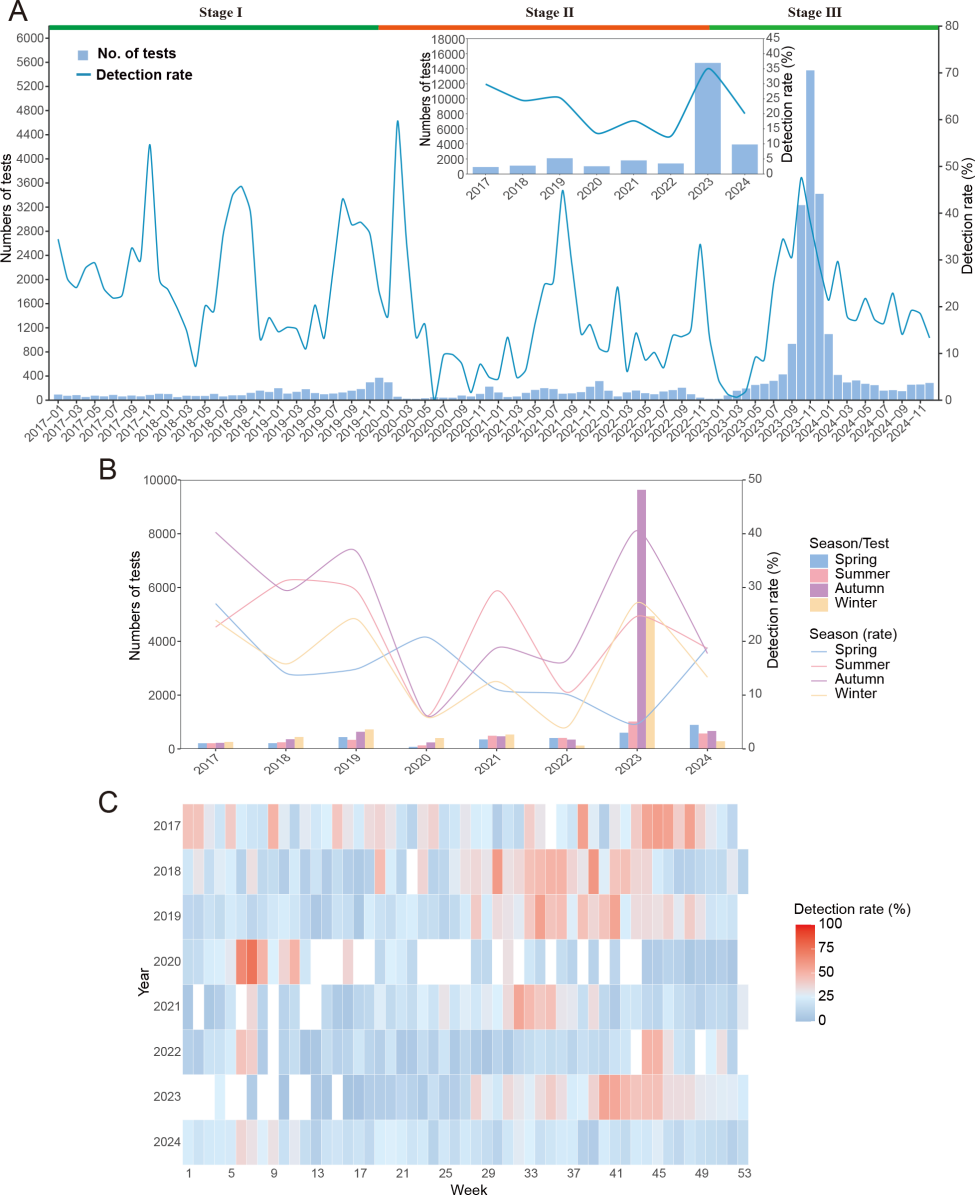
Epidemiological characteristics and seasonal variations of *M. pneumoniae* in 27,056 patients who presented with respiratory infectious diseases (RIDs) from 1 January 2017 to 31 December 2024. (**A**) Monthly distribution of tested samples for (bars) and detection rates of (lines), Stage I: from 1 January 2017 to 31 December 2019; Stage II: from 1 January 2020 to 31 December 2022; Stage III: from 1 January 2023 to 31 December 2024. (**B**) Yearly distribution of the *M. pneumoniae* rate in different seasons. (**C**) Heatmaps of *M. pneumoniae* positivity rates by week from 1 January 2017 to 31 December 2024.

The average detection rate for *M. pneumoniae* was 28.36% (7,672/27,056), with rates fluctuating from a low of 12.56% (176/1,401) in 2022 to a high of 35.01% (5,173/14,774) in 2023. Prior to 2020, a distinct epidemic pattern in *M. pneumoniae* outbreaks among children was observed, with peak detection rates predominantly occurring between August and November. An exception was noted in February 2020, which presented an unusually high detection rate of 59.65% (34/57). Notably, there was a significant decrease in the percentage of *M. pneumoniae*-positive samples from 2020 to 2022. The rates were 13.53% (139/1,027), 17.65% (319/1,807), and 12.56% (176/1,401), respectively. The average detection rate remained consistently low at 14.97% (634/4,235), with some fluctuations observed on a monthly basis. Particularly in June 2020, the positivity rate plummeted to almost 0. From 1 January 2023 to 31 December 2024, following the easing of NPIs, there was a remarkable increase in the number of children with RIDs undergoing tests, peaking at 54.61% (14,774/27,056) in 2023. Furthermore, the highest positivity detection rate of 47.57% (1,538/3,233) was recorded in October 2023. After this spike in the epidemic, the number of children with RIDs began to decline each month from January 2024 to 31 December 2024, with the average positivity rate dropping to an average level of 20.09% (789/3,928) during that timeframe (Fig. 1A; Table S1).

A notable disparity was observed in the infection rates of *M. pneumoniae* across various seasons (*P* < 0.001). The infection rate was highest in autumn at 36.71% (4, 618/12,578), followed by winter at 23.12% (1,782/7,707), summer at 22.75% (777/3,415), and spring at 13.92% (444/3,190). Additionally, there were changes in the timing of peak infection seasons for *M. pneumoniae*; for example, in both 2018 and 2021, the peak occurred during the summer, whereas in 2020, an out-of-season spring was the peak-infection season, and in 2024, the peak occurred during the spring. (Fig. 1B).

The heatmap visually illustrates the seasonal patterns of *M. pneumoniae* among the overall samples. The positivity rate on a weekly basis for *M. pneumoniae* revealed changes in both the prevalence and the timing of the annual peak. Before 2020, the activity of *M. pneumoniae* was mainly observed between the 28th and 40th weeks. Conversely, from 2020 to 2022, the epidemic displayed an unusual and abbreviated duration, occurring outside the normal season. In 2023, the most recent epidemic commenced at approximately the 28th week and concluded near the 48th week, and the epidemic lasted for approximately 20 weeks. Different from previous years, *M. pneumoniae* in 2024 demonstrated significantly reduced duration, persisting for merely 10 weeks during the first quarter of the year (Fig. 1C).

### Age distributions of *M. pneumoniae* infection in children

**Fig 2:**
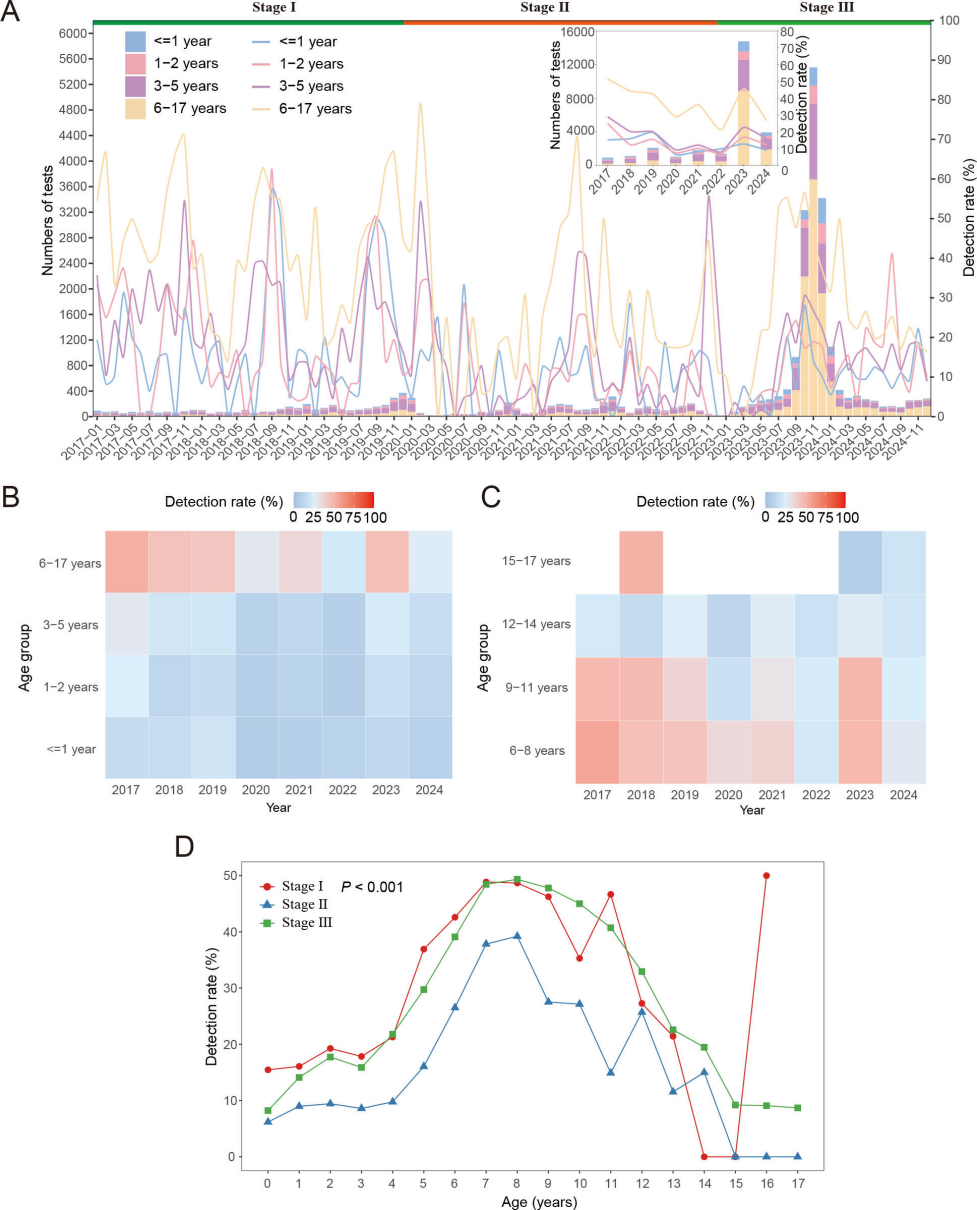
*M. pneumoniae* positivity rates in different age groups. (**A**) Monthly distribution of tested samples for (bars) and detection rates of (lines) by age. Stage I: from 1 January 2017 to 31 December 2019; Stage II: from 1 January 2020 to 31 December 2022; Stage III: from 1 January 2023 to 31 December 2024. (**B**) Heatmap of the percentage of *M. pneumoniae*-positive individuals between 0 and 17 years of age during different years. (**C**) Heatmap of the percentage of *M. pneumoniae*-positive individuals between 6 and 17 years of age during different years. (**D**) *M. pneumoniae* positivity rates in different age groups during the three stages.

There was a significant difference in the *M. pneumoniae* infection rate among children in different age groups (*P* < 0.001) (Table 1). The highest infection rate was observed in the 6–17 years at 41.08% (5,332/12,980), followed by the 3–5 years at 18.65% (1,635/8,766), the 1–2 years at 14.25% (363/2,548), with the lowest rate in the ≤1 year at 12.38% (342/2,762). Notably, in the years 2018, 2019, and 2022, the detection rate in the ≤1 year was higher than that in the 1–2 years (2018: 15.89% vs 12.28%; 2019: 19.59% vs 15.48%; 2022: 9.80% vs 7.14%) (Fig. 2A; Table S1).

The age span within the 6–17 years was relatively large. To enhance our understanding of the age distribution, we further subdivided this group into four categories: 6–8 years, 9–11 years, 12–14 years, and 15–17 years. Heatmaps revealed that the *M. pneumoniae* infection rate increased with age from 6 to 8 years, followed by a decrease in the infection rate from ages 9–17 years. Notably, the positivity rate was highest in the 6–8 years (Fig. 2B and C), and 8 years of age could be seen as a turning point for *M. pneumoniae* infection rates for the three stages (Fig. 2D).

### Macrolide-resistant mutation analysis of *M. pneumoniae*

**Fig 3:**
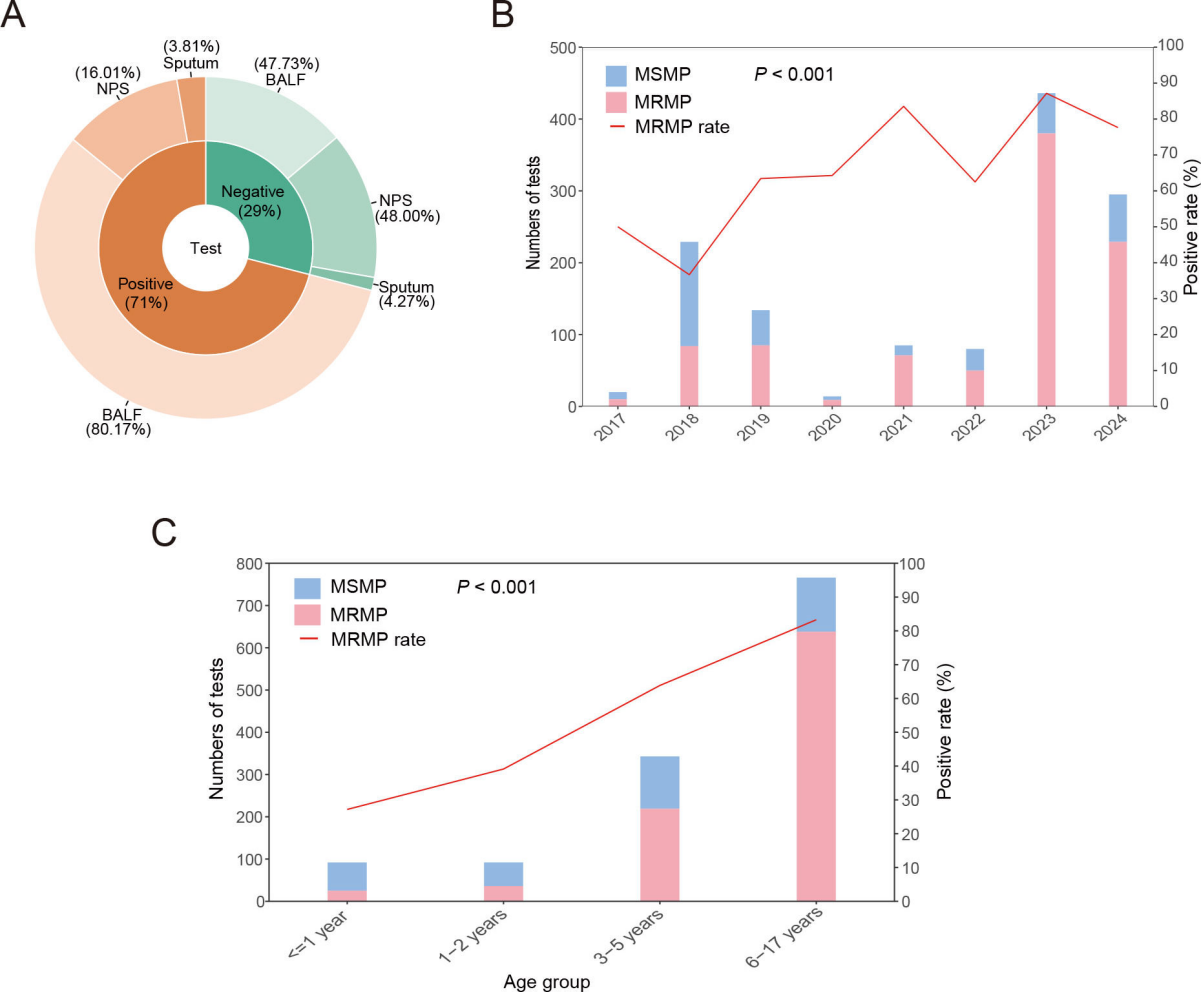
Characteristics of macrolide resistance in *M. pneumoniae* (MRMP) in 1,293 patients who presented with respiratory infectious diseases (RIDs) from 1 January 2017 to 31 December 2024. (**A**) Overall MRMP positivity rate and sample type rate. BALF: bronchoalveolar lavage fluid, NPS: nasopharyngeal swabs. (**B**) MRMP positivity rates across different years. (**C**) MRMP positivity rates across different age groups.

In this investigation, MRMP tests were analyzed in a cohort of 1,293 patients. Of these, 71.00% (918/1,293) tested positive. The positivity rates showed no statistically significant difference between genders, with 69.16% (453/655) detected in males and 72.88% (465/638) in females (*P* = 0.140) (Table 3). Of these MRMP-positive cases, the samples included 80.17% (736/918) from BALF, 16.01% (147/918) from NPS, and 3.81% (35/918) from sputum (Fig. 3A; Table S2). Between 1 January 2017 and 31 December 2024, the annual rate of MRMP varied from 36.68% (84/229) in 2018 to 87.16% (380/436) in 2023 (Fig. 3B; Table S3).

**TABLE 3:** Basic characteristics of the overall macrolide-resistance-mutations samples (*n* = 1,293)

Variables	Total (n)	Positive tests (n)	MRMP rate (%)^*a*^*^,^*^*b*^	Statistic	P
Sex	(*n* = 1,293)			χ²=2.18	0.140
Male	655	453	69.16		
Female	638	465	72.88		
Age group, y	(*n* = 1,293)			χ²=195.91	<0.001
≤ 1	92	25	27.17		
1–2	92	36	39.13		
3–5	343	219	63.85		
6–17	766	638	83.29		
Season	(*n* = 1,293)			χ²=76.27	<0.001
Spring	221	109	49.32		
Summer	260	195	75.00		
Autumn	448	364	81.25		
Winter	364	250	68.68		

^*a*^ MRMP: macrolide-resistance mutations of *M. pneumoniae.*

^*b*^ MRMP rate (%) was expressed as the positive number/the total number (%).

Significant differences in MRMP rates were found across various age groups (*P* < 0.001). The highest positivity rate was recorded in the 6–17 years (83.29%), followed by those aged 3–5 years (63.85%), those aged 1–2 years (39.13%), and those aged ≤1 year (27.17%) (Fig. 3C).

### Impact of the COVID-19 pandemic and the effects of NPIs on the prevalence of *M. pneumoniae*

In Stage I, the detection rate of *M. pneumoniae* was 26.12%, which significantly decreased to 14.97% in Stage II (*P* < 0.001). This rate then increased to a higher level of 31.88% in Stage III (*P* < 0.001).

The detection rate of *M. pneumoniae* during Stage II was analyzed in comparison to that of Stage I and Stage III across all samples, as well as in various groups, defined by gender, age, and season. A significant difference was observed between Stage II and Stage III, except for the spring and summer season (11.51% vs 13.12%, *P* = 0.261; 18.93% vs 22.50%, *P* = 0.029) and ≤1 year (8.25% vs 11.84%, *P* = 0.024).

For *M. pneumoniae*, we conducted a further examination of the differences between stage I and stage III characteristics. In the age category, only children who were aged ≤1 year exhibited a notable decrease of 5.19% in stage III compared with stage I (11.84% vs 17.03%, *P* < 0.001). However, the rates of positivity for *M. pneumoniae* among other age groups at stage III were similar to those for stage I (1–2 years: 15.61% vs 16.61%, *P* = 0.572; 3–5 years: 20.88% vs 21.68%, *P* = 0.477; 6–17 years: 42.11% vs 44.68%, *P* = 0.105). In terms of gender, there was a significant increase in the positivity rates within this group compared with those in stage I patients (male: 31.99% vs 25.90%, *P* < 0.001; female: 31.74% vs 26.41%, *P* < 0.001).

Interestingly, a statistically significant increase was noted in autumn and winter for Stage III compared with Stage I (autumn: 39.10% vs 35.24%, *P* = 0.009; winter: 25.96% vs 20.30%, *P* < 0.001). In contrast, a minor decrease was recorded in spring and summer in Stage III in relation to Stage I (13.12% vs 17.63%, *P* = 0.003; 22.50% vs 28.12%, *P* = 0.002) (Table 2).

The analysis revealed a significant increase in MRMP rates across the three stages, with values of 46.74%, 72.63%, and 83.31%, respectively (*P* < 0.001). We assessed the impact of the COVID-19 pandemic and the subsequent easing of NPIs on MRMP incidence across various subgroups, including gender, age, and season (Table S4).

## DISCUSSION

*M. pneumoniae* is a prevalent respiratory pathogen associated with RIDs in China. It can also lead to severe clinical manifestations, particularly affecting the cardiovascular, hematologic, and nervous systems, as well as the skin, and, in some cases, may even result in death (17–19). Enhancing the precision of early diagnosis and preventing the onset of severe pneumonia is essential. Although culturing *M. pneumoniae* is regarded as the gold standard for diagnosis, the demanding growth conditions and slow proliferation rate make this approach impractical for its detection. Conversely, identifying the nucleic acids of *M. pneumoniae*, such as its DNA or RNA, provides high sensitivity and specificity, making it an effective method for the early identification of infections caused by *M. pneumoniae* (20). It is essential to explore how the pandemic and subsequent modifications in public health strategies have influenced the transmission and occurrence of *M. pneumoniae* infections. Gaining insights into these dynamics can offer a critical understanding of the relationship between respiratory pathogens and public health initiatives amid a global health emergency.

Our investigation tracked 27,056 children who were diagnosed with RIDs and underwent *M. pneumoniae*-DNA tests or MRMP tests over an 8-year timeframe from 1 January 2017 to 31 December 2024. This research examines the three phases of the COVID-19 pandemic (pre-pandemic, pandemic, and post-pandemic) and assesses the epidemiological features of *M. pneumoniae* within Henan Province for the first time.

*M. pneumoniae* infections occur throughout the year in various climates and regions globally, with epidemic cycles occurring every 1 to 3 years, and the most recent occurrences of *M. pneumoniae* were recorded in 2013 and 2016. It can be expected that the subsequent epidemic could have extended from the autumn and winter of 2019 through the winter and spring of 2020 (4, 21, 22). In our research, prior to the COVID-19 outbreak in China (2017–2019), *M. pneumoniae* epidemic peaks occurred primarily between August and November during the summer and autumn months, with an average positivity rate of 26.12%, while they fluctuated slightly each year within a range of 24.46% to 29.86%. We found a periodic variation in infection prevalence between August and November 2019, with rates oscillating between 35.97% and 42.97%. Following previous trends, this ongoing figure is potentially foreshadowing an extended epidemic.

The latest epidemic took place from late 2019 to early 2020 and affected multiple countries, primarily Europe and Asia (11, 23). However, following the emergence of COVID-19 in December 2019, China implemented extensive NPIs (such as mask-wearing, social distancing, and improving hand hygiene) alongside “dynamic COVID-zero” strategies to mitigate the spread of SARS-CoV-2. As a result, in addition to limiting COVID-19 transmission, there have been significant changes in the incidence of other respiratory pathogens, including influenza, parainfluenza, RSV, as well as *M. pneumoniae*, due to these stringent measures (24, 25).

Our research indicates that since February 2020, the positive detection rate of *M. pneumoniae* during the COVID-19 pandemic (2020–2022) has experienced a substantial decline compared with that in the pre-pandemic period (2017–2019). This rate stabilized at a lower level of 14.97%, in contrast to the pre-pandemic rate of 26.12%, with some fluctuations observed throughout the pandemic, indicating a nonseasonal epidemic that persisted for nearly 3 years. These findings align with those of earlier studies suggesting that the positivity rate of *M. pneumoniae* was lower than that in the pre-COVID-19 era (23, 26–29). Overall, NPIs significantly curtailed the transmission of *M. pneumoniae*. First, due to *M. pneumoniae*’s aerosol-based transmission (particle size 0.5–5 µm) and its designation as one of the smallest self-replicating organisms (0.1–0.3 µm in diameter), mask usage reduces droplet/aerosol concentrations in inhaled air (30). Second, social distancing interrupts person-to-person transmission networks, lowering the “basic reproduction number,” particularly in high-density environments such as schools (31). Third, improved hand hygiene eliminates pathogens to some extent.

In December 2022, China announced further enhancements to its measures for preventing and controlling the COVID-19 epidemic, progressively relaxing NPIs. Following the removal of NPI policies, China has experienced an increase in respiratory infections, including influenza viruses, RSV, and *M. pneumoniae*.

Beginning in June 2023, numerous areas in China experienced an early surge in *M. pneumoniae* infections among children. By September 2023, there was a notable increase in cases of *M. pneumoniae* infection, accompanied by severe clinical symptoms (15, 16). In contrast to the earlier resurgence of other respiratory pathogens following the cessation of NPIs, the re-emergence of *M. pneumoniae* occurred notably later than the discontinuation of these measures (32, 33). A similar pattern was observed in our study; the number of patients with RIDs and the positivity rate of *M. pneumoniae* significantly increased beginning in August, peaking in October at 47.57%. Notably, the overall positivity rate of *M. pneumoniae* in 2023 reached its highest level at 35.01%. The phenomenon known as “delayed emergence” can be elucidated through various theoretical frameworks. Waning herd immunity, commonly referred to as “immune debt,” leads to a deficiency in protective immunity, thereby increasing individual susceptibility to infectious diseases.

During a gap of up to 3 years while NPIs were in place, children experienced limited exposure to pathogens, which impacted their developing immunity and resulted in a relative weakening of herd immunity. Furthermore, transient herd immunity following the last epidemic period from late 2019 to early 2020, coupled with the extended duration of NPIs, created a potential risk for more intense outbreaks of respiratory pathogens. Additional contributing factors may include the introduction of new subtypes into the population, as well as the characteristics of *M. pneumoniae*, which include a slow generation time (approximately 6 hours), a lengthy incubation period (ranging from 1 to 3 weeks), and a relatively low rate of transmission. These factors may lead to a prolonged interval necessary for the reconsolidation of *M. pneumoniae* infections within a population (34–36). The 2023 autumn outbreak may signify a resurgence of the cyclical *M. pneumoniae* epidemics observed before the onset of the COVID-19 pandemic. Our findings align with those of recent studies indicating a worldwide increase in *M. pneumoniae* infections, particularly from July 2023 onward (14, 33, 37–39).

Following the notable increase in RIDs and *M. pneumoniae* infections from August to November 2023, there was a gradual decline in the number of *M. pneumoniae* tests conducted. During 2024, the positivity rate fell to an average of 20.09%. This decline indicates that the epidemic has nearly reverted to levels comparable to the pre-pandemic rate of 26.12%. Ongoing surveillance is still necessary to understand the prevalence of *M. pneumoniae*.

Previous studies have shown that *M. pneumoniae* infections may occur in different seasons, and the positivity rate may peak in summer, autumn, or winter (19, 23, 40). Our study revealed that infections attributed to *M. pneumoniae* exhibit a distinct seasonal pattern characterized by a primary peak in autumn, followed by peaks in winter, whereas spring has the lowest prevalence. However, the peak season for *M. pneumoniae* infections may have varied slightly from year to year throughout the duration of the research. The optimal growth temperature for *M. pneumoniae* is between 36°C and 37°C. In China, July, August, and September are the hottest months of the year, which coincides with favorable conditions for the growth of *M. pneumoniae*. Autumn marks the back-to-school season in China, during which children congregate in closed or semi-closed air-conditioned environments that may facilitate the transmission of *M. pneumoniae*. Additionally, extremely cold and hot weather may influence individuals’ immune functions, contributing to a gradual increase in the rate of *M. pneumoniae* infections as the lowest temperatures increase.

Throughout the pandemic, effective control strategies ensured a consistently low rate of *M. pneumoniae* positivity, and seasonality was not clearly evident. However, as NPIs were relaxed, our monitoring indicates that the *M. pneumoniae* epidemic in 2023 displayed a distinct seasonal trend, peaking in the autumn and winter. The interval between the previous epidemic in 2019 and the current outbreak was approximately 4 years, which generally aligns with the typical occurrence of *M. pneumoniae* outbreaks every 3 to 7 years (21).

Throughout the study duration, the infection rate of *M. pneumoniae* varied among different age groups, with the highest prevalence observed in the 6–17 years followed by the 3–5 years, and the ≤1 year presenting the lowest prevalence. Research conducted by Cheng et al., Zhang et al., and Jiang et al. supports this finding (23, 28, 38). In contrast, our study revealed a different positivity rate of *M. pneumoniae* than earlier investigations did, which indicated that the detection of *M. pneumoniae* was more frequent in children aged 3 years (26, 41). Additionally, our research indicated a notable decline in *M. pneumoniae* cases starting at age 8. The discrepancies between our findings and those of other studies may stem from variations in detection methodologies and the populations involved, as well as the ongoing maturation of children’s immune systems and the diminishing effects of maternal immunity, both of which may interact to influence this outcome (42). During the COVID-19 pandemic, a significant reduction in positivity rates was observed across all age categories compared with both the pre-pandemic and post-pandemic periods. Improved public health measures have effectively curtailed the transmission of *M. pneumoniae* among individuals of all ages.

The global prevalence of MRMP has been increasing, particularly in Asian countries, whereas Europe and the United States report significantly lower detection rates of MRMP (6, 43, 44). In mainland China, MRMP rates range from 69% to 100%, with the A2063G mutation in the 23S rRNA region being the most frequently observed mutation (10, 45–47). Our research revealed an average MRMP rate of 71.00%. Notably, the MRMP rate during the pre-pandemic phase was 46.74%. In contrast, during the COVID-19 pandemic, despite rigorous adherence to NPIs, we documented an increase in MRMP prevalence to 72.63% compared with pre-pandemic levels. The post-pandemic MRMP rate significantly increased to 83.31% relative to the earlier phases.

The consistent upward trend in MRMP may be associated with the overuse of macrolide antibiotics for treating *Mycoplasma pneumoniae pneumonia* (MPP) in children as these drugs remain the first-line treatment for this condition in China (48). Notably, the highest positive MRMP rate occurred among children aged 6–17 years and during autumn, which corresponds with data regarding *M. pneumoniae* by age and season. MRMP typically presents with symptoms such as high fever, severe coughing, and impaired mental health, leading to prolonged illness, extended hospital stays, and poor prognoses. Reports indicate that the overall antibiotic usage rate in children with CAP in China is 89.08%, highlighting the urgent need for appropriate antibiotic therapies in clinical settings (49).

Our surveillance findings provide valuable insights into the prevalence of *M. pneumoniae*. Notably, the positivity rates for *M. pneumoniae* and MRMP during the post-pandemic period increased significantly compared with those in the earlier two time frames. This observation suggests a potential correlation between the increase in common respiratory infections among children and the relaxation of public health measures. Consequently, it is imperative to maintain a robust public health management strategy to prevent the resurgence of severe infectious diseases following the COVID-19 pandemic.

This study has several limitations. First, although we analyzed data from a substantial sample size obtained from the largest maternal and child health hospital in Henan Province, it is important to acknowledge that this research was conducted at a single center; consequently, the conclusions drawn may not fully reflect the overall provincial landscape. Second, our investigation was restricted to mutations at positions A2063G and A2064G within domain V of the 23S rRNA gene, which may lead to an underestimation of the rate of macrolide resistance. Third, our surveillance relied exclusively on PCR-based detection of *M. pneumoniae* DNA, the current diagnostic standard. As serological tests (e.g., IgM/IgG) were not used due to their limited sensitivity and specificity, we cannot fully rule out potential contributions of the “immunity debt” hypothesis to the pathogen’s resurgence, which might require seroconversion data for validation. Finally, given the limited sample size prior to 2023 and the inherent challenges of subgroup analyses—particularly in year- and age-stratified MRMP trends—the reduced statistical power due to smaller sample sizes highlights the need for extending the observation period, gathering more comprehensive clinical data, and conducting multicenter studies to strengthen and validate the findings.

## Data Availability

The data used in this study are available upon request. Due to privacy and confidentiality concerns, the original contributions presented in the study are included in the article/supplementary material, and further inquiries can be directed to the corresponding authors.

## SUPPLEMENTAL MATERIAL

Supplemental tables spectrum.03121-24-s0001.docx Tables S1 to S4.

ASM does not own the copyrights to Supplemental Material that may be linked to, or accessed through, an article. The authors have granted ASM a non-exclusive, world-wide license to publish the Supplemental Material files. Please contact the corresponding author directly for reuse.
