# Supplementary material for: Epidemiological changes of Mycoplasma pneumoniae among children before, during, and post the COVID-19 pandemic in Henan, China, from 2017 to 2024
Source: Microbiol Spectr. 2025 Jun 9;13(7):e03121-24. doi: 10.1128/spectrum.03121-24 (PMC12252384; doi:10.1128/spectrum.03121-24)
Supplement: Supplemental tables — Tables S1 to S4. [file spectrum.03121-24-s0001.docx]

**Supplementary materials**

**Supplementary Table 1.**

**Basic characteristics of *M.pneumoniae* in 27,056 patients presenting with respiratory infectious diseases from 1 January 2017 to 31 December 2024.**

|  | Total | 2017 | 2018 | 2019 | 2020 | 2021 | 2022 | 2023 | 2024 |
| --- | --- | --- | --- | --- | --- | --- | --- | --- | --- |
|  | No. Pos/no. Tested （Detection rate, %） | | | | | | | | |
| PCR testing |  |  |  |  |  |  |  |  |  |
| Overall | 7,672/27,056 (28.36) | 275/921 (29.86) | 271/1,108 (24.46) | 530/2,090 (25.36) | 139/1,027 (13.53) | 319/1,807 (17.65) | 176/1,401 (12.56) | 5,173/14,774 (35.01) | 789/3,928 (20.09) |
| Age group, y |  |  |  |  |  |  |  |  |  |
| ≤ 1 | 342/2,762 (12.38) | 37/244 (15.16) | 24/151 (15.89) | 48/245 (19.59) | 8/124 (6.45) | 19/232 (8.19) | 15/153 (9.80) | 154/1,207 (12.76) | 37/406 (9.11) |
| 1-2 | 363/2,548 (14.25) | 33/132 (25.00) | 21/171 (12.28) | 50/323 (15.48) | 12/161 (7.45) | 25/248 (10.08) | 12/168 (7.14) | 168/1,001 (16.78) | 42/344 (12.21) |
| 3-5 | 1,635/8,766 (18.65) | 97/335 (28.96) | 102/506 (20.16) | 189/949 (19.92) | 45/487 (9.24) | 102/846 (12.06) | 49/609 (8.05) | 845/3,741 (22.59) | 206/1,293 (15.93) |
| 6-17 | 5,332/12,980 (41.08) | 108/210 (51.43) | 124/280 (44.29) | 243/573 (42.41) | 74/255 (29.02) | 173/481 (35.97) | 100/471 (21.23) | 4,006/8,825 (45.39) | 504/1,885 (26.74) |
| Sex |  |  |  |  |  |  |  |  |  |
| Male | 4,229/14,949(28.29) | 156/531 (29.38) | 157/608 (25.82) | 290/1,189 (24.39) | 78/579 (13.47) | 183/1,046 (17.50) | 90/760 (11.84) | 2,858/8,088 (35.34) | 417/2,148(19.41) |
| Female | 3,443/12,107 (28.44) | 119/390 (30.51) | 114/500 (22.80) | 240/901 (26.64) | 61/448 (13.62) | 136/761 (17.87) | 86/641 (13.42) | 2,315/6,686 (34.62) | 372/1,780 (20.90) |
| Season |  |  |  |  |  |  |  |  |  |
| Spring | 444/3,190 (13.92) | 57/211 (27.01) | 30/213 (14.08) | 65/438 (14.84) | 16/77 (20.78) | 39/352 (11.08) | 41/405 (10.12) | 28/602 (4.65) | 168/892 (18.83) |
| Summer | 777/3,415 (22.75) | 48/212 (22.64) | 77/246 (31.30) | 99/336 (29.46) | 8/131 (6.11) | 144/490 (29.39) | 43/409 (10.51) | 251/1,018 (24.66) | 107/573 (18.67) |
| Autumn | 4,618/12,578 (36.71) | 91/226 (40.27) | 106/360 (29.44) | 234/637 (36.73) | 15/242 (6.20) | 88/469 (18.76) | 57/346 (16.47) | 3,909/9,633 (40.58) | 118/665 (17.74) |
| Winter | 1,782/7,707(23.12) | 62/259 (23.94) | 70/443 (15.80) | 175/725 (24.14) | 24/404 (5.94) | 67/535 (12.52) | 5/123 (4.07) | 1,341/4,933 (27.18) | 38/285 (13.33) |

Detection rate (%) were expressed as the positive number/the total number (%)

**Supplementary Table2.**

**Overall MRMP positivity rate and sample type rate**

| Sample type | Total (n) (%) | Positive tests (n) (%) | MRMP rate (%) |
| --- | --- | --- | --- |
|  | (n = 1293) | (n = 918) |  |
| BALF | 915 (70.77) | 736 (80.17) | 56.92 |
| NPS | 327 (25.29) | 147 (16.01) | 11.37 |
| Sputum | 51 (3.94) | 35 (3.81) | 2.71 |

MRMP: macrolide-resistance mutations M. pneumoniae, BALF: bronchoalveolar lavage fluid, NPS: nasopharyngeal swabs.

**Supplementary Table3.**

**Basic characteristics of MRMP in 1,293 patients presenting with respiratory infectious diseases from 1 January 2017 to 31 December 2024.**

|  | Total | 2017 | 2018 | 2019 | 2020 | 2021 | 2022 | 2023 | 2024 |
| --- | --- | --- | --- | --- | --- | --- | --- | --- | --- |
|  | No. Pos/no. Tested （MRMP rate, %） | | | | | | | | |
| PCR testing |  |  |  |  |  |  |  |  |  |
| Overrall | 918/1,293 (71.00) | 10/20 (50.00) | 84/229 (36.68) | 85/134 (63.43) | 9/14 (64.29) | 71/85 (83.53) | 50/80 (62.50) | 380/436 (87.16) | 229/295 (77.63) |
| Age group, y |  |  |  |  |  |  |  |  |  |
| ≤ 1 | 25/92 (27.17) | 1/5 (20.00) | 4/33 (12.12) | 3/11 (27.27) | - | 2/4 (50.00) | 2/5 (40.00) | 7/15 (46.67) | 6/19 (31.58) |
| 1-2 | 36/92 (39.13) | 0/1 (0) | 7/35 (20.00) | 5/14 (35.71) | - | 4/4 (100.00) | 1/3 (33.33) | 7/14 (50.00) | 12/21 (57.14) |
| 3-5 | 219/343 (63.85) | 5/8 (62.50) | 33/87 (37.93) | 29/50 (58.00) | 3/5 (60.00) | 17/20 (85.00) | 10/21 (47.62) | 59/76 (77.63) | 63/76 (82.89) |
| 6-17 | 638/766 (83.29) | 4/6 (66.67) | 40/74 (54.05) | 48/59 (81.36) | 6/9 (66.67) | 48/57 (84.21) | 37/51 (72.55) | 307/331 (92.75) | 148/179 (82.68) |
| Gender |  |  |  |  |  |  |  |  |  |
| Male | 453/655 (69.16) | 4/9 (44.44) | 43/117 (36.75) | 41/67 (61.19) | 3/5 (60.00) | 37/45 (82.22) | 26/42 (61.90) | 194/226 (85.84) | 105/144 (72.92) |
| Female | 465/638 (72.88) | 6/11 (54.55) | 41/112 (36.61) | 44/67 (65.67) | 6/9 (66.67) | 34/40 (85.00) | 24/38 (63.16) | 186/210 (88.57) | 124/151 (82.12) |
| Season |  |  |  |  |  |  |  |  |  |
| Spring | 109/221 (49.32) | - | 13/90 (14.44) | 15/29 (51.72) | 1/1 (100.00) | 11/13 (84.62) | 17/21 (80.95) | 8/11 (72.73) | 44/56 (78.57) |
| Summer | 195/260 (75.00) | - | 21/38 (55.26) | 26/43 (60.47) | - | 34/38 (89.47) | 14/25 (56.00) | 53/58 (91.38) | 47/58 (81.03) |
| Autumn | 364/448 (81.25) | - | 26/53 (49.06) | 17/19 (89.47) | 2/3 (66.67) | 18/22 (81.82) | 8/20 (40.00) | 249/276 (90.22) | 44/55 (80.00) |
| Winter | 250/364 (68.68) | 28/56 (50.00) | 25/45 (55.56) | 12/18 (66.67) | 4/5 (80.00) | 14/20 (70.00) | 3/6 (50.00) | 150/189 (79.37) | 14/25 (56.00) |

MRMP rate (%) were expressed as the positive number/the total number (%)

MRMP: macrolide-resistance mutations M. pneumoniae

**Supplementary Table4.**

**Comparison of positive rate of MRMP in three stages according to the COVID-19 epidemic status**

| Group | Stage Ⅰ  (2017-2019) | | Stage Ⅱ  (2020-2022) | | Stage Ⅲ  (2023-2024) | | Stage Ⅰ vs Stage Ⅱ | | Stage Ⅱ vs Stage Ⅲ | | Stage Ⅰ vs Stage Ⅲ | |
| --- | --- | --- | --- | --- | --- | --- | --- | --- | --- | --- | --- | --- |
|  | No. Pos/no. Tested | MRMP rate, % | No. Pos/no. Tested | MRMP rate, % | No. Pos/no. Tested | MRMP rate, % | Statistic | *P value* | Statistic | *P value* | Statistic | *P value* |
| PCR testing |  |  |  |  |  |  |  |  |  |  |  |  |
| Overrall | 179/383 | 46.74 | 130/179 | 72.63 | 609/731 | 83.31 | χ²=33.03 | <0.001 | χ²=10.76 | 0.001 | χ²=162.41 | <0.001 |
| Age group, y |  |  |  |  |  |  |  |  |  |  |  |  |
| ≤ 1 | 8/49 | 16.33 | 4/9 | 44.44 | 13/34 | 38.24 | χ²=3.66 | 0.056 | χ²=0.12 | 0.735 | χ²=5.10 | 0.024 |
| 1-2 | 12/50 | 24.00 | 5/7 | 71.43 | 19/35 | 54.29 | χ²=6.60 | 0.010 | χ²=0.70 | 0.403 | χ²=8.15 | 0.004 |
| 3-5 | 67/145 | 46.21 | 30/46 | 65.22 | 122/152 | 80.26 | χ²=5.05 | 0.025 | χ²=4.48 | 0.034 | χ²=37.19 | <0.001 |
| 6-17 | 92/139 | 66.19 | 91/117 | 77.78 | 455/510 | 89.22 | χ²=4.19 | 0.041 | χ²=11.07 | 0.001 | χ²=43.73 | <0.001 |
| Gender |  |  |  |  |  |  |  |  |  |  |  |  |
| Male | 88/193 | 45.60 | 66/92 | 71.74 | 299/370 | 80.81 | χ²=17.14 | <0.001 | χ²=3.66 | 0.056 | χ²=73.20 | <0.001 |
| Female | 91/190 | 47.89 | 64/87 | 73.56 | 310/361 | 85.87 | χ²=15.95 | <0.001 | χ²=7.70 | 0.006 | χ²=90.62 | <0.001 |
| Season |  |  |  |  |  |  |  |  |  |  |  |  |
| Spring | 28/119 | 23.53 | 29/35 | 82.86 | 52/67 | 77.61 | χ²=40.83 | <0.001 | χ²=0.39 | 0.534 | χ²=51.15 | <0.001 |
| Summer | 47/81 | 58.02 | 48/63 | 76.19 | 100/116 | 86.21 | χ²=5.21 | 0.022 | χ²=2.86 | 0.091 | χ²=20.00 | <0.001 |
| Autumn | 43/72 | 59.72 | 28/45 | 62.22 | 293/331 | 88.52 | χ²=0.07 | 0.788 | χ²=21.94 | <0.001 | χ²=35.38 | <0.001 |
| Winter | 61/111 | 54.95 | 25/36 | 69.44 | 164/217 | 75.58 | χ²=2.35 | 0.125 | χ²=0.61 | 0.433 | χ²=14.50 | <0.001 |

The Bonferroni correction α is *P* < 0.05/3, which corresponds to *P* < 0.0167
